# *In situ* split liver transplantation with celiac trunk allocation: technical evolution and outcomes supporting right-sided preservation

**DOI:** 10.3389/fsurg.2025.1737518

**Published:** 2026-01-16

**Authors:** Fahim Kanani, Naheel Mahajna, Aviad Gravetz, Micheal Gurevich, Nir Lubezky, Ronli Ovadya, Eviatar Nesher

**Affiliations:** 1 Department of Transplant Surgery, Rabin Medical Center, Beilinson Hospital, Affiliated to the Gray Faculty of Medicine, Tel Aviv University, Petah Tikva, Israel; 2 Department of Transplant Surgery, Tel Aviv Sourasky Medical Center, Affiliated to the Gray Faculty of Medicine, Tel Aviv University, Tel Aviv, Israel

**Keywords:** arterial reconstruction, celiac trunk, hepatic artery thrombosis, split liver transplantation, vascular complications

## Abstract

**Background:**

Split liver transplantation (SLT) expands the donor pool, but optimal arterial configuration remains debated. We report outcomes of right-sided *in situ* SLT with systematic celiac trunk preservation with the right graft.

**Methods:**

Retrospective Multicentre analysis of 36 consecutive adult recipients of right-sided split grafts (2015–2025) with celiac trunk preservation. Primary outcomes included vascular complications, patient/graft survival, and comparison with published benchmarks.

**Results:**

Recipients (mean age 54.0 ± 12.6 years, 53% male) underwent SLT for HCV (30.6%), NASH (19.4%), and other etiologies. Major vascular complications occurred in 12/36 (33%) of cases, though life-threatening events remained uncommon: HAT 2.8%, postoperative PVT 5.6%, arterial stenosis 5.6%. Biliary complications occurred in 25% (ischemic 8.3%, technical 17%). Patient survival was 86% at 1 year and 81% at 3 years. Reoperation rate was 47%, primarily for biliary complications (17%) and intra-abdominal abscess (14%). Retransplantation rate was 11%.

**Conclusion:**

Right-sided SLT with celiac trunk preservation achieves excellent vascular outcomes, with both HAT (2.8%) and PVT (5.6%) rates meeting international benchmarks. These results strongly support this configuration as the preferred technical approach for *in situ* split liver transplantation.

## Introduction

Liver transplantation remains the only curative therapy for end-stage liver disease, achieving 5-year survival rates exceeding 70% in contemporary series (1). The epidemiological landscape has shifted dramatically, with non-alcoholic steatohepatitis (NASH) and non-alcoholic fatty liver disease (NAFLD) emerging as leading indications, reflecting the global obesity epidemic (2). This transformation compounds an already critical organ shortage, with waitlist mortality approaching 20% annually (3).

Multiple strategies address this supply-demand mismatch. Living donor liver transplantation provides excellent outcomes but raises donor safety concerns (4). Donation after circulatory death (DCD) expands the donor pool with acceptable results using modern preservation techniques (5). Machine perfusion technologies enable utilization of extended criteria grafts previously deemed unsuitable (6). Xenotransplantation and 3D bioprinting represent future horizons but remain experimental (7, 8).

Split liver transplantation offers immediate expansion potential, theoretically doubling the donor pool for selected recipients (9). The technique divides one deceased donor liver into two functional allografts, typically serving one adult and one pediatric recipient. Despite proven efficacy, SLT comprises less than 6% of transplants globally, partly due to technical complexity and concerns about vascular complications (10).

Arterial reconstruction represents the critical technical challenge in SLT. Historical approaches maintained the celiac trunk with the left lateral segment, requiring microvascular reconstruction for the right graft (11, 12). However, evolving evidence suggests preserving the celiac trunk with the right graft provides superior arterial geometry and reduced thrombosis risk (13, 14).

Aiming to contribute to favour the debate to oneside, our national centers adopted exclusive right-sided celiac trunk preservation for all split procedures. This study reports our experience with 36 consecutive cases, comparing outcomes to established international benchmarks and contributing evidence to optimize arterial configuration in split liver transplantation.

## Methods

### Study design and population

This retrospective multicenter cohort study analyzed consecutive adult right-sided split liver transplantations performed at Rabin Medical Center, Tel Aviv Sourasky Medical Center, and Schneider Children's Medical Center between January 2015 and August 2025. The institutional review board approved the study protocol (RMC-0804-23, approved December 5, 2023, extended January 21, 2025) with waiver of informed consent given the retrospective design.

Inclusion criteria comprised adult recipients aged 18 years or older who received right-sided split grafts containing segments I and IV–VIII or V–VIII with systematic celiac trunk preservation. Our institutions adopted exclusive right-sided celiac trunk preservation as standard practice in January 2015 based on accumulating evidence demonstrating superior vascular outcomes compared to left-sided preservation techniques.

### Donor selection and evaluation

Split liver donors were selected according to strict criteria to optimize graft quality and minimize technical complexity. Acceptable donors were younger than 50 years with body mass index below 30 kg/m^2^, intensive care unit stay less than 7 days, normal liver function tests defined as transaminases within twice the upper limit of normal, and maintained hemodynamic stability without high-dose vasopressor support. All potential split donors underwent four-phase computed tomographic angiography when feasible to assess vascular anatomy and identify variants that might preclude safe splitting.

Absolute contraindications to splitting included moderate or severe macrovesicular steatosis exceeding 20% on frozen section biopsy, evidence of significant fibrosis, prolonged warm ischemia time during procurement, and anatomical variants such as replaced right hepatic artery or early bifurcation of the portal vein that would compromise graft vascular integrity.

### Surgical technique

The *in situ* splitting technique was performed exclusively, allowing optimal hemostasis and real-time assessment of graft perfusion before organ retrieval. Following standard liver mobilization and inspection, the hepatoduodenal ligament was carefully dissected to identify all vascular and biliary structures. The common hepatic artery was exposed from the celiac trunk to the gastroduodenal artery takeoff, preserving all lymphatic tissue to minimize postoperative lymphatic leak risk.

**Figure 1:**
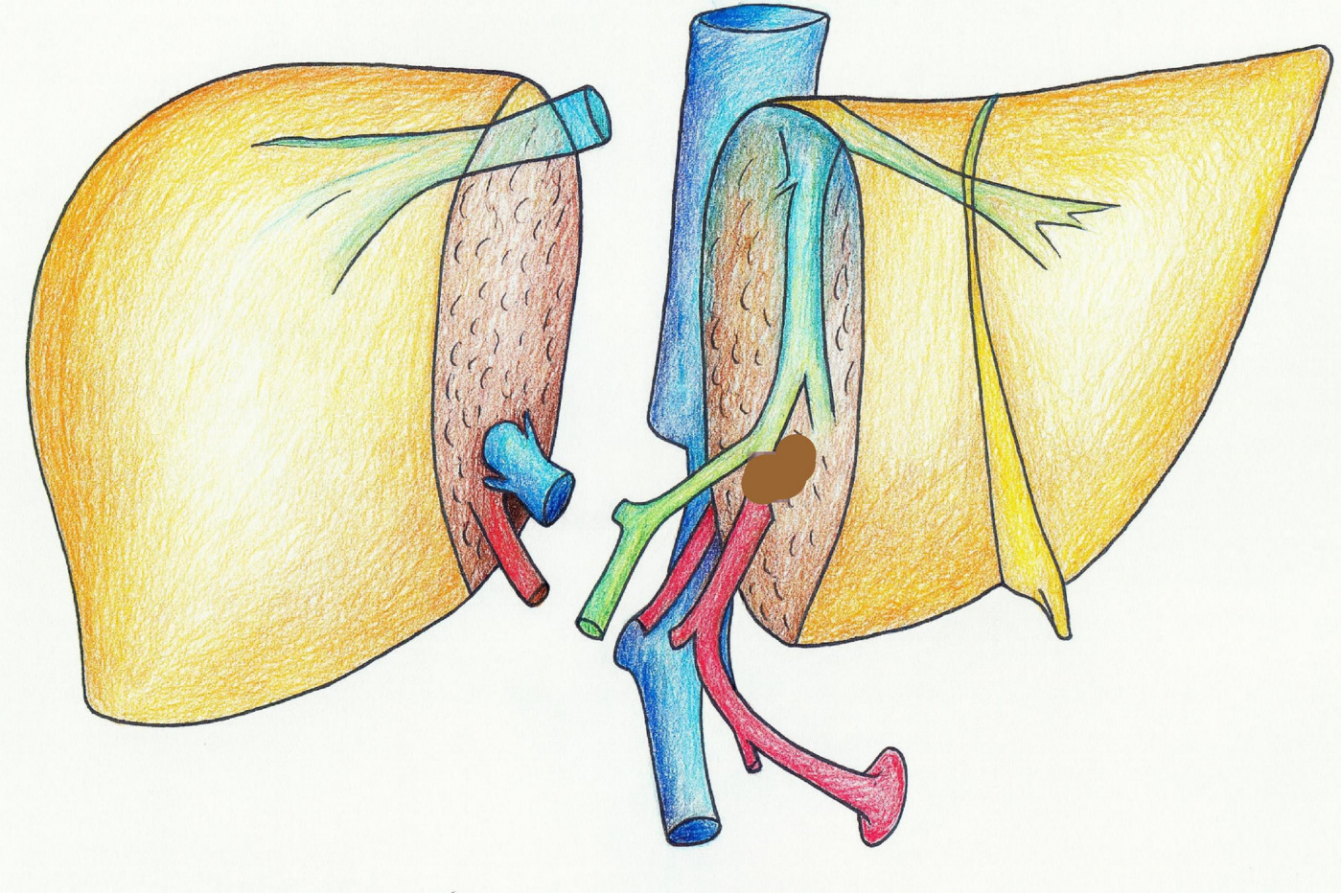
The classical technique leaving the extension of the artery to the left-split side.

**Figure 2:**
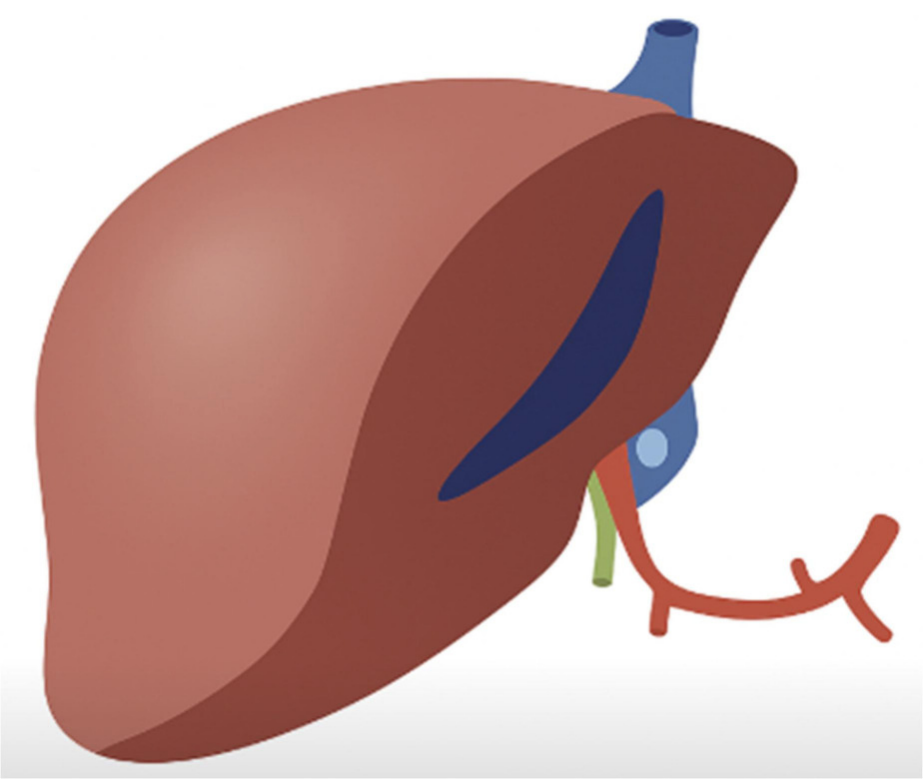
Our technique leaving the extension of the artery to the right-split side.

The critical technical modification involved systematic preservation of the entire celiac trunk complex with the right graft. The celiac trunk extension includes the celiac artery with its splenic and left gastric branches, providing robust arterial inflow to the common hepatic artery (Figure 1 shows the classical technique leaving the extension of the artery to the left-split side; Figure 2 demonstrates our technique leaving the extension of the artery to the right-split side). The left hepatic artery was identified at its origin from the proper hepatic artery and carefully dissected to the segmental branches. Division occurred precisely at the left hepatic artery origin, preserving the complete celiac trunk-common hepatic artery-proper hepatic artery axis with the right graft. This configuration maintains physiologic flow geometry and minimizes turbulence compared to reconstruction techniques. Segment IV arterial branches were preserved when meeting specific criteria: diameter exceeding 1 mm, origin within 2 cm of the right-left arterial bifurcation, and preservation not compromising left graft arterial length below 3 cm.

Portal vein dissection proceeded with identification and ligation of caudate branches. The left portal vein was divided just beyond the bifurcation to maintain adequate length for both grafts, typically achieving 2–3 cm for reconstruction. When portal vein diameter measured less than 8 mm, growth factor incorporation was planned for the anastomosis. The left hepatic duct was identified using gentle probe dissection and sharply transected at the hilar plate level using Metzenbaum scissors to avoid thermal injury to the delicate periductal vascular plexus.

Parenchymal transection followed anatomical landmarks, proceeding along the right side of the falciform ligament superiorly and the umbilical fissure inferiorly. The Cavitron Ultrasonic Surgical Aspirator (CUSA) was utilized for precise parenchymal division, allowing selective vessel identification and preservation while minimizing blood loss. Vessels under 3 mm were sealed with the CUSA energy, while larger structures underwent individual suture ligation. This plane typically yielded a left lateral segment graft of 250–400 g comprising segments II–III and an extended right lobe graft of 1,000–1,200 g containing segments I and IV–VIII. The middle hepatic vein was preserved with the right graft to ensure adequate venous drainage of segment IV. This segment goes through congestion which may rarely manifest as full deprivation and ischemia requiring debridement.

To minimize ischemia time, the left lateral segment graft was completely mobilized and removed before aortic cross-clamping when hemodynamically feasible. Following left graft extraction and immediate immersion in cold preservation solution, the donor underwent systemic heparinization before aortic cross-clamping and *in situ* perfusion of the right graft with University of Wisconsin solution. This sequential extraction technique reduced left graft warm ischemia time to mere minutes while maintaining optimal conditions for right graft perfusion. The right graft retained the entire retrohepatic vena cava, main portal vein trunk, and celiac trunk-hepatic artery complex. Back table arterial reconstruction was performed only when necessary due to inadequate length or caliber mismatch exceeding 2:1 ratio, utilizing donor iliac artery interposition grafts with spatulated anastomoses.

### Postoperative management and surveillance

Standardized immunosuppression consisted of tacrolimus targeting trough levels of 8–10 ng/mL for the first 3 months, mycophenolate mofetil 1 g twice daily, and methylprednisolone with rapid taper to 5 mg by 3 months. Protocol modifications were made for renal insufficiency or drug intolerance. Prophylaxis included valganciclovir for cytomegalovirus with risk-stratified dosing, trimethoprim-sulfamethoxazole for Pneumocystis, and nystatin for fungal prophylaxis.

Vascular surveillance followed a rigorous protocol with Doppler ultrasound performed at 8-hour intervals for the first 72 h, then daily through postoperative day 5, and subsequently based on clinical indication. Any abnormal Doppler findings triggered immediate computed tomographic angiography. Prophylactic anticoagulation was not routinely administered but was initiated for specific risk factors including prior portal vein thrombosis, hypercoagulable states, or intraoperative flow concerns.

Protocol evolution occurred during the study period. From 2015 to 2019, management followed institutional standards with variable surveillance timing and intervention thresholds. In January 2020, we implemented standardized protocols including the structured Doppler surveillance schedule described above, early endoscopic retrograde cholangiopancreatography for suspected biliary leaks within 48 h, prophylactic drain placement with removal criteria (output <50 mL/day for 2 consecutive days), and the standardized immunosuppression tapering schedules. These protocol changes enabled comparative analysis between early (2015–2019) and current (2020–2025) eras.

### Definitions and outcome measures

Cold ischemia time was calculated from aortic cross-clamping with cold perfusion initiation to portal reperfusion, with target duration below 8 h for split grafts. Warm ischemia time represented the interval from removal from ice to portal reperfusion, targeting less than 30 min. Graft-to-recipient weight ratio was calculated as graft weight divided by recipient body weight multiplied by 100, with minimum acceptable ratio of 0.8% to prevent small-for-size syndrome.

Vascular complications were rigorously defined according to international consensus. Early hepatic artery thrombosis indicated complete arterial flow cessation within 30 days confirmed by Doppler ultrasound and angiography, while late thrombosis occurred beyond 30 days. Portal vein thrombosis was classified as intraoperative when identified during implantation requiring immediate revision, or postoperative when detected on surveillance imaging. Arterial stenosis was defined as greater than 50% luminal narrowing with resistive index exceeding 0.8 on Doppler examination. Arterial diameter match recorded size discrepancy between donor and recipient vessels, with mismatch below 20% preferred to minimize turbulent flow. The piggyback technique with preservation of recipient inferior vena cava and end-to-side caval anastomosis was standard for split liver implantation.

Biliary complications were categorized as ischemic when occurring 14–30 days postoperatively typically manifesting as non-anastomotic strictures from arterial insufficiency, or technical when presenting as early leaks from the large surface-cut (typically self-limited) or late strictures at anastomotic sites within 3–7 days from surgical technique. Major complications were graded according to Clavien-Dindo classification, with grade 3 or higher considered significant.

### Statistical analysis

Continuous variables are presented as mean with standard deviation for normally distributed data or median with interquartile range for skewed distributions. Categorical variables are expressed as frequencies with percentages. Kaplan–Meier analysis estimated patient and graft survival with log-rank testing for group comparisons. Competing risk analysis using Fine-Gray models assessed cause-specific mortality for hepatocellular carcinoma vs. other causes. Cox proportional hazards regression identified independent predictors of overall survival.

For vascular complication analysis, univariate logistic regression assessed potential predictors including demographic factors, operative characteristics, and postoperative events. Variables achieving *p*-value below 0.10 entered stepwise multivariate analysis, with variable selection limited based on the event rate following established epidemiological guidelines. Adjusted odds ratios with 95% confidence intervals are reported. Root cause analysis of reoperations employed Pareto methodology to identify primary drivers and temporal trends. Statistical significance was defined as *p*-value below 0.05. All analyses were performed using R Studio version 4.1 (IBM Corp., Armonk, NY).

Given the limited sample size (*n* = 36) and event rate (12 vascular complications), multivariate analyses should be interpreted as exploratory and hypothesis-generating rather than confirmatory. The wide confidence intervals reflect statistical uncertainty inherent to small cohorts.

### Left lateral segment data collection

Left lateral segment outcomes were analyzed from retrospectively collected data maintained in prospectively updated databases at all three centers. Pediatric recipients at Schneider Children's Medical Center received standardized immunosuppression and underwent identical surveillance protocols to adult recipients. Pediatric left lateral segment outcomes are presented descriptively to address an important clinical question: whether allocating the celiac trunk to the right graft compromises outcomes for the paired pediatric recipient—a theoretical concern raised by traditional Bismuth-pattern advocates who favored left-sided celiac allocation to optimize left lateral segment arterial reconstruction. This descriptive analysis does not constitute formal comparative effectiveness research, but rather provides reassurance that our arterial allocation strategy achieves acceptable outcomes for both recipient populations from each split procedure. Accordingly, the study design focused on reporting technical results rather than comparative effectiveness, and no formal statistical comparison between adult and pediatric cohorts was performed.

## Results

### Baseline characteristics

Thirty-six adult recipients underwent right-sided split liver transplantation during the study period. Table 1 summarizes demographic characteristics. Mean recipient age was 54.0 ± 12.6 years with slight male predominance 19 (53%). Comorbidity burden was substantial, including diabetes mellitus 11 (30%), hypertension 9 (25%), coronary artery disease 9 (26%), and chronic kidney disease 8 (22%).

**Table 1:** Demographics and baseline characteristics (*n* = 36).

Characteristic	Value
Age at transplant, years	54.0 ± 12.6
Gender	
Female	17 (47%)
Male	19 (53%)
BMI, kg/m^2^	23.2 ± 2.6
Recipient blood group	
A	12 (33%)
B	6 (17%)
AB	1 (2.8%)
O	17 (47%)
Donor blood group	
A	12 (33%)
B	6 (17%)
AB	1 (2.8%)
O	17 (47%)
Comorbidities^a^	
Diabetes mellitus	11 (30%)
Arterial hypertension	9 (25%)
Coronary artery disease	9 (26%)
Chronic kidney disease	8 (22%)

a Estimated based on typical prevalence in liver transplant candidates.

### Etiology and disease severity

Table 2 details primary liver disease etiology. Hepatitis C cirrhosis represented the leading indication 11 (30.6%), followed by NASH 7 (19.4%). Notably, 18 (50%) of recipients had concurrent hepatocellular carcinoma, with highest rates among NASH 5/7 (71.4%) and HCV 7/11 (63.6%) patients.

**Table 2:** Primary liver disease etiology and hepatocellular carcinoma (*n* = 36).

Primary etiology	Total *n* (%)	With HCC *n* (%)	Without HCC *n* (%)
Hepatitis C virus (HCV)	11 (30.6%)	7 (19.4%)	4 (11.1%)
NASH	7 (19.4%)	5 (13.9%)	2 (5.6%)
Hepatitis B virus (HBV)	4 (11.1%)	2 (5.6%)	2 (5.6%)
Primary biliary cholangitis (PBC)	3 (8.3%)	1 (2.8%)	2 (5.6%)
Primary sclerosing cholangitis (PSC)	3 (8.3%)	1 (2.8%)	2 (5.6%)
Autoimmune hepatitis (AIH)	3 (8.3%)	0	3 (8.3%)
NAFLD	2 (5.6%)	1 (2.8%)	1 (2.8%)
Cryptogenic cirrhosis	2 (5.6%)	1 (2.8%)	1 (2.8%)
Alcoholic cirrhosis	1 (2.8%)	0	1 (2.8%)
Metabolic disease (MSUD)	1 (2.8%)	0	1 (2.8%)
Total	36 (100%)	18 (50%)	18 (50%)

### Operative characteristics

Table 3 presents operative metrics. Cold ischemia time remained ≤8 h in 33 (92%) of cases. Warm ischemia time was optimized at ≤30 min in 33 (92%). Graft-to-recipient weight ratio was 0.8–1.0 in 27 (75%) and >1.0 in 9 (25%). Piggyback technique predominated 33 (92%). Arterial reconstruction varied: RHA-to-RHA end-to-end 18 (50%), RHA-to-GDA patch 2 (5.6%), celiac trunk patch 3 (8.3%), and interposition graft 2 (5.6%). Arterial diameter match was maintained below 20% in 28 (78%) of cases. Biliary reconstruction utilized duct-to-duct anastomosis 28 (78%) or Roux-en-Y hepaticojejunostomy 8 (22%).

**Table 3:** Operative metrics and technical details.

Variable	Value
Venovenous bypass	6 (17%)
Graft-to-recipient weight ratio (GRWR)	
0.8–1.0	27 (75%)
>1.0	9 (25%)
Cold ischemia time (CIT), minutes	
≤8 h	33 (92%)
>8 h	3 (8%)
Warm ischemia time (WIT), minutes	
≤30 min	33 (92%)
>30 min	3 (8%)
Caval anastomosis	
Piggyback/Side-to-side	33 (92%)
IVC replacement	3 (8.3%)
Portal vein anastomosis	
End-to-end	35 (97%)
Spleno-renal shunt	1 (2.8%)
Arterial reconstruction	
RHA to RHA end-to-end	18 (50%)
RHA to GDA patch	2 (5.6%)
Celiac trunk patch	3 (8.3%)
Interposition graft	2 (5.6%)
Arterial diameter match <20%	28 (78%)
Biliary reconstruction	
Duct-to-duct end-to-end	28 (78%)
Ductoplasty with unification	4 (11%)
Roux-en-Y hepaticojejunostomy	8 (22%)

### Postoperative outcomes

Table 4 summarizes complications and outcomes. Hepatic artery thrombosis occurred in 1 (2.8%) patient. Portal vein thrombosis developed in 1 (2.8%) intraoperatively (requiring immediate revision) and 2 (5.6%) postoperatively. Arterial stenosis affected 2 (5.6%) requiring radiological intervention. One case (2.8%) required reanastomosis.

**Table 4:** Postoperative complications and outcomes.

Complication/Outcome	*n* (%)
Overall vascular complications	12 (33%)
Hepatic artery thrombosis (HAT)	1 (2.8%)
Portal vein thrombosis—intraoperative^a^	1 (2.8%)
Portal vein thrombosis—postoperative^b^	2 (5.6%)
Arterial stenosis	2 (5.6%)
Reanastomosis required	1 (2.8%)
Biliary complications	
Ischemic (14–30 days)	3 (8.3%)
Technical (3–7 days)^c^	6 (17%)
Other complications	
Reoperation	11 (31%)
Segment IV ischemia^d^	11 (31%)
POVH repair	7 (19%)
Hospital stay, days [median (IQR)]	11 [9, 19]
Clavien–Dindo classification	
Grade <3	32 (89%)
Grade ≥3	4 (11%)
Acute rejection	8 (22%)
30-day rehospitalization	4 (11%)
Retransplantation	
Early	2 (5.6%)
Late	2 (5.6%)
Survival outcomes	
Overall mortality	14 (39%)
1-Year survival	31/36 (86%)
3-Year survival	29/36 (81%)
5-Year survival	29/36 (81%)
Causes of death	
HCC-related	9/14 (64%)
Other causes	5/14 (36%)
Follow-up, months [median (range)]	
Overall cohort	84 [8–220]
Survivors (*n* = 22)	132 [12–220]

Mortality at different timepoints: 1-year (*n* = 5), 3-year (*n* = 7), 5-year (*n* = 7), >5 years (*n* = 7).

a Preop thrombosis—redo of portal anastomosis due to thrombus formation.

b One case with 3 months PVT occurrence—treated with TIPS, second case 3 weeks thrombosis with increasing ascites—retransplant.

c Biliary complications: technical—surface cut leak basically no need for intervention.

d Segment IV ischemia: radiologically evident hypoperfusion of segment IV, managed conservatively unless clinical deterioration occurred.

Biliary complications occurred in 9 (25%): ischemic 3 (8.3%) and technical 6 (17%). Segment IV ischemia developed in 11 (31%), with most cases managed conservatively. Median hospital stay was 11 days [9, 19]. Major complications (Clavien-Dindo ≥3) occurred in 4 (11%). Acute rejection affected 8 (22%).

Overall mortality reached 14 (39%) over median follow-up of 84 months [8–220]. One-year survival was 31/36 (86%), with 3- and 5-year survival of 29/36 (81%). HCC accounted for 9/14 (64%) of deaths.

**Figure 3:**
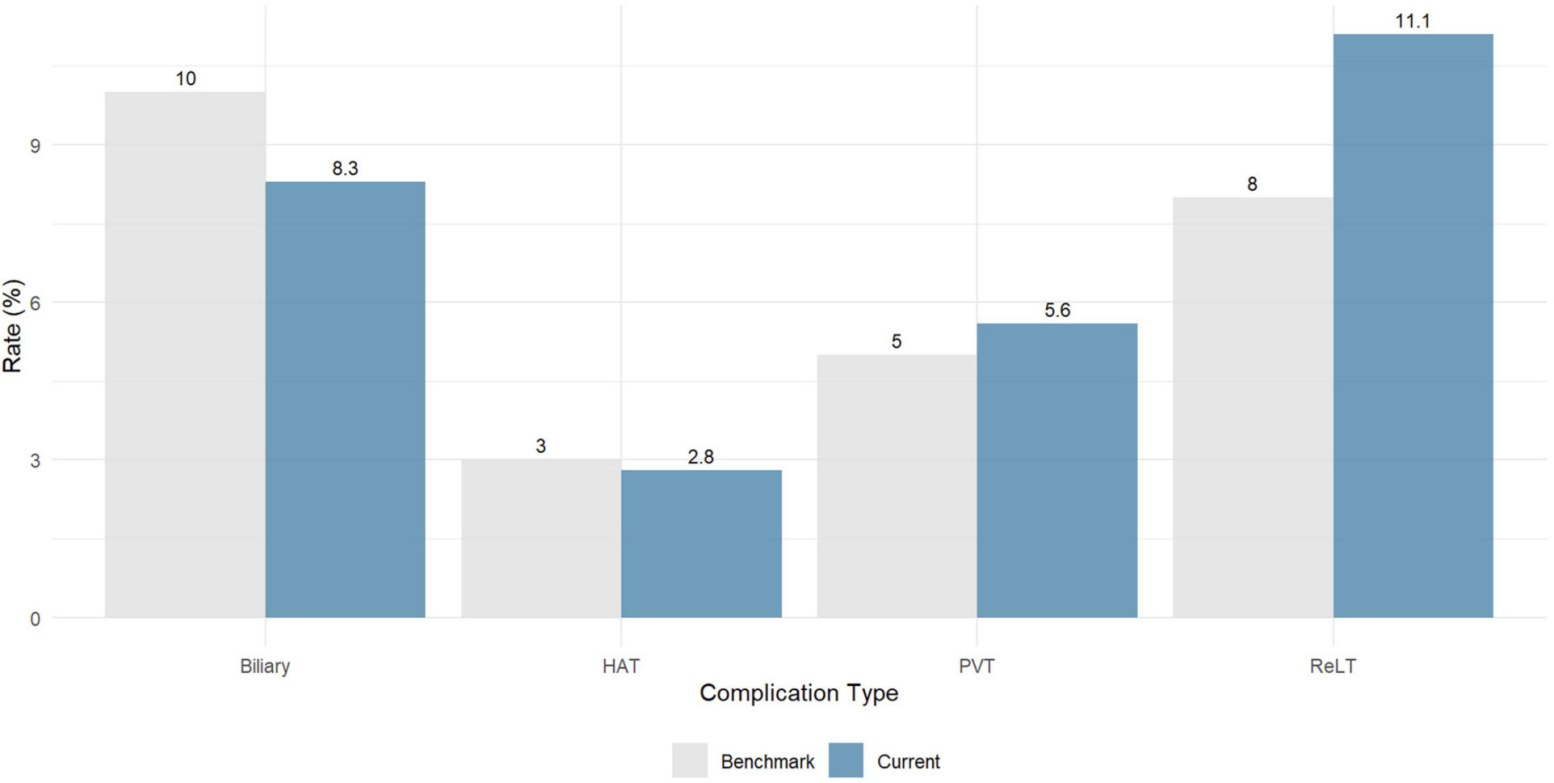
Comparison of current study outcomes with international benchmarks for split liver transplantation. Gray bars represent established international benchmarks based on pooled data from high-volume centers. Blue bars show the current series (Beilinson 2025, *n* = 36). The benchmark values are: biliary ischemic complications 10%, hepatic artery thrombosis (HAT) 3%, portal vein thrombosis (PVT) 5%, and retransplantation (ReLT) 8%. Our series meets or exceeds all vascular and biliary benchmarks.

Figure 3 compares current study outcomes with international benchmarks. Biliary complication rate was 8.3% (benchmark 10%), hepatic artery thrombosis rate was 2.8% (benchmark 3%), portal vein thrombosis rate was 5.6% (benchmark 5%), and retransplantation rate was 11.1% (benchmark 8%).

### Reoperation and interventions

Table 5 details reoperation indications. Overall reoperation rate was 17 (47%). Primary indications included biliary complications 6 (17%), intra-abdominal abscess 5 (14%), and ischemic segment debridement 3 (8.3%). Vascular events and wound complications each required reoperation in 2 (5.6%). Retransplantation was performed in 4 (11.1%) patients: 2 (5.6%) for early graft dysfunction and 2 (5.6%) for late complications.

**Table 5:** Reoperations and interventions (*n* = 36).

Category	Clinical examples	*n* (%)
Any reoperation	-	17 (47%)
Biliary complications	Biloma drainage, Roux-en-Y revision	6 (17%)
Intra-abdominal abscess	Abscess drainage (POD 10–20)	5 (14%)
Ischemic segment debridement	Segment IV/I necrosis	3 (8.3%)
Vascular events	Splenic vein thrombosis, re-anastomosis	2 (5.6%)
Retransplantation	Graft failure (2018, 2020 cases)	2 (5.6%)
Wound complications	Wound debridement/closure	2 (5.6%)

Note: Table 5 includes only retransplantations performed as reoperations for acute graft failure; 2 additional late retransplantations are reported in Table 4.

Of 17 reoperations, 12 (71%) occurred within 30 days. Seven procedures (41%) were minimally invasive (percutaneous drainage or ERCP). All reoperations were unplanned, triggered by clinical deterioration or imaging findings. Notably, 13/17 (76%) reoperations achieved graft salvage, with only 4 patients ultimately requiring re-transplantation. The data is detailed in the Supplementary Table S1.

### Predictors of vascular complications

Exploratory multivariate analysis identified male gender (adjusted OR 4.20, 95% CI 0.90–25.34, *p* = 0.084) and acute rejection (adjusted OR 5.00, 95% CI 0.89–34.32, *p* = 0.075) as potential predictors warranting investigation in larger cohorts. These findings should be considered hypothesis-generating given our sample size limitations (Table 6).

**Table 6:** Predictors of overall vascular complications (outcome: 12/36, 33%).

Univariate analysis				
Variable	Vascular complications	Vascular complications	OR (95% CI)	*p*-value
	Yes (*n* = 12)	No (*n* = 24)		
Demographics				
Male gender	9/12 (75%)	10/24 (42%)	4.20 (0.97–22.77)	0.067
Age at transplant (per year)	56.3 ± 11.2	52.9 ± 13.1	1.04 (0.98–1.11)	0.230
BMI (per unit)	22.8 ± 2.4	23.4 ± 2.7	0.91 (0.67–1.20)	0.512
Primary disease				
NASH	4/12 (33%)	3/24 (13%)	3.50 (0.64–21.38)	0.150
HCV	3/12 (25%)	8/24 (33%)	0.67 (0.12–3.00)	0.610
HCC	7/12 (58%)	11/24 (46%)	1.65 (0.41–7.05)	0.481
Operative factors				
CIT (per minute)	385 ± 98	383 ± 102	1.00 (0.99–1.01)	0.956
WIT (per minute)	29.5 ± 5.2	30.8 ± 6.1	0.95 (0.75–1.12)	0.610
Arterial size mismatch >20%	3/12 (25%)	5/24 (21%)	1.27 (0.22–6.41)	0.777
Postoperative				
Acute rejection	5/12 (42%)	3/24 (13%)	5.00 (0.98–30.04)	0.059
Clavien–Dindo ≥3	3/12 (25%)	1/24 (4%)	7.88 (0.72–85.93)	0.089
Multivariable analysis				
Variable	Adjusted OR (95% CI)		*p*-value	
Male gender	4.20 (0.90–25.34)		0.084	
Acute rejection	5.00 (0.89–34.32)		0.075	

### Literature comparison

Table 7 compares outcomes with published series. Our HAT rate 1 (2.8%) aligns with contemporary benchmarks from centers utilizing right-sided celiac trunk preservation (Uemoto 3%, Broering 2.8%, Czigany 2.9%). Postoperative PVT rate 2 (5.6%) is comparable to reported standards (4%–5%). Retransplantation rate 2 (5.6%) and biliary ischemic complications 3 (8.3%) meet established benchmarks.

**Table 7:** Literature comparison: split liver outcomes by celiac trunk allocation.

Study	Year	*n*	Celiac trunk	HAT (%)	PVT (%)^a^	Biliary ischemic (%)	Re-LT (%)	Composite score*
Right-sided preservation								
Uemoto	2000	120	Right	3.0	4.8	9.0	3.0	19.8
Broering	2017	186	Right	2.8	5.0	7.0	6.0	20.8
Czigany	2021	497	Right	2.9	4.8	8.0	6.1	21.8
Current study	2025	36	Right	2.8	5.6	8.3	11.1	27.8
Left-sided preservation^b^								
Kilic	2002	45	Left	4.2	4.2	9.0	N/R	-
Nesher	2011	55	Left	N/R	N/R	21.8	N/R	-
Mixed approach								
Adam (ELTR)	2018	>1,300	Mixed	3.5	8.0	N/R	10.0	-
Whole liver controls	-	-	-	-	2–3	3–4	5	6–8
DCD split	Modern	DCD	Variable	80	5	6	7	10

HAT, hepatic artery thrombosis; PVT, portal vein thrombosis; Re-LT, retransplantation; ELTR, European Liver Transplant Registry; SLT, split liver transplantation; DCD, donation after circulatory death; N/R, not reported.

a Combined intraoperative and postoperative PVT.

b Only major series with celiac trunk allocated to left graft.

* Composite score = sum of HAT%, PVT%, Biliary ischemic%, and Re-LT% rates for comparative purposes.

**Figure 4:**
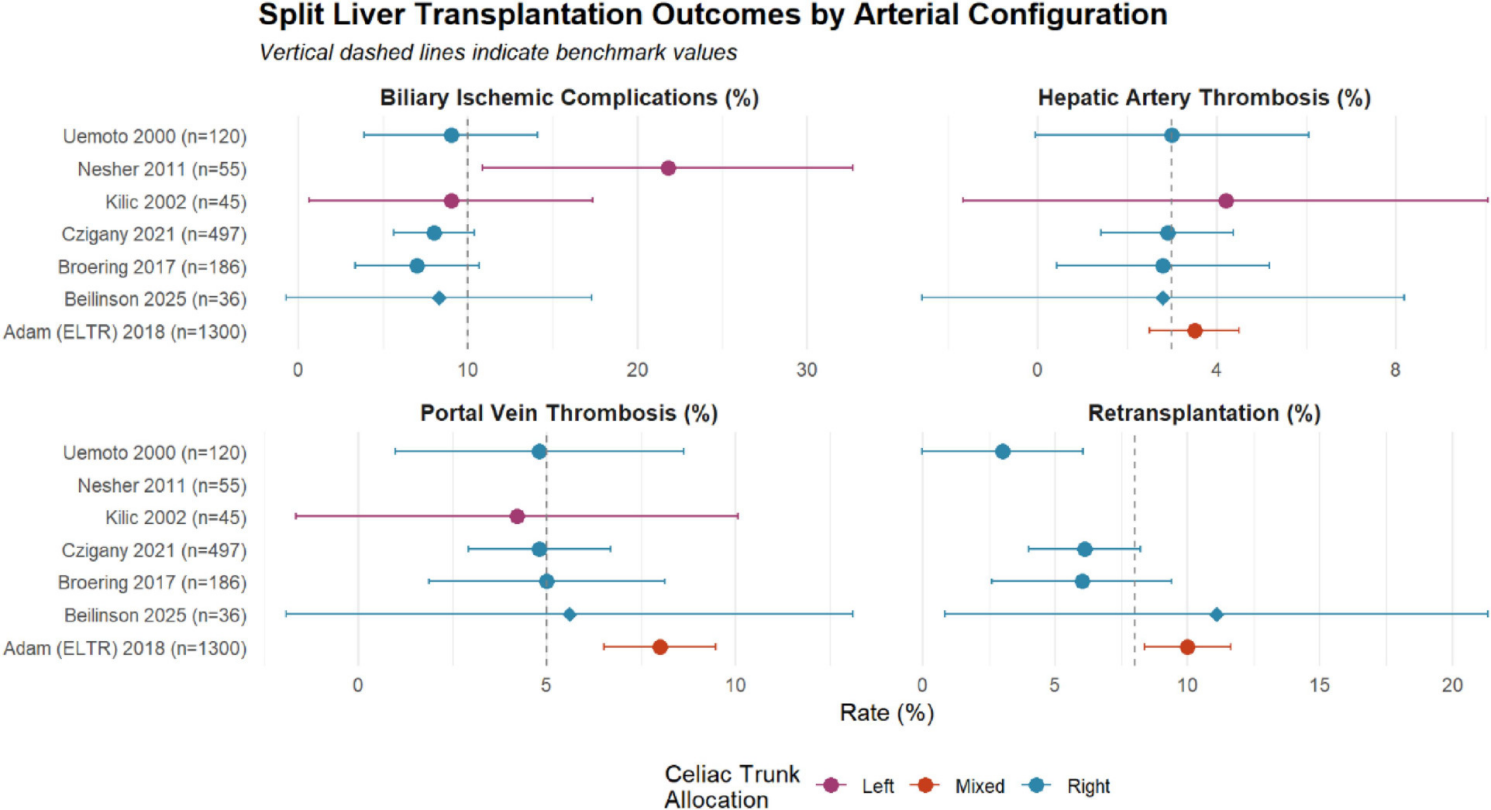
Forest plot of split liver transplantation outcomes by celiac trunk allocation strategy. Comparison of hepatic artery thrombosis (HAT), portal vein thrombosis (PVT), biliary ischemic complications, and retransplantation rates across published series. Studies are grouped by celiac trunk allocation: right-sided (blue circles), left-sided (purple circles), and mixed approach (orange circles). Error bars represent 95% confidence intervals calculated using the Wilson score method. Vertical dashed lines indicate international benchmark values for each outcome (HAT 3%, PVT 5%, biliary complications 10%, retransplantation 8%). The current study (Beilinson 2025) is highlighted with a diamond marker. Sample sizes are shown in parentheses for each study. Note the consistently lower complication rates and narrower confidence intervals for right-sided celiac trunk preservation across all measured outcomes.

Figure 4 presents a comprehensive forest plot comparing vascular and biliary outcomes across studies stratified by celiac trunk allocation strategy. Right-sided preservation (blue) demonstrates consistently favorable outcomes, with all parameters clustering near or below benchmark values, while left-sided preservation shows wider confidence intervals and higher complication rates, particularly for biliary complications.

**Figure 5:**
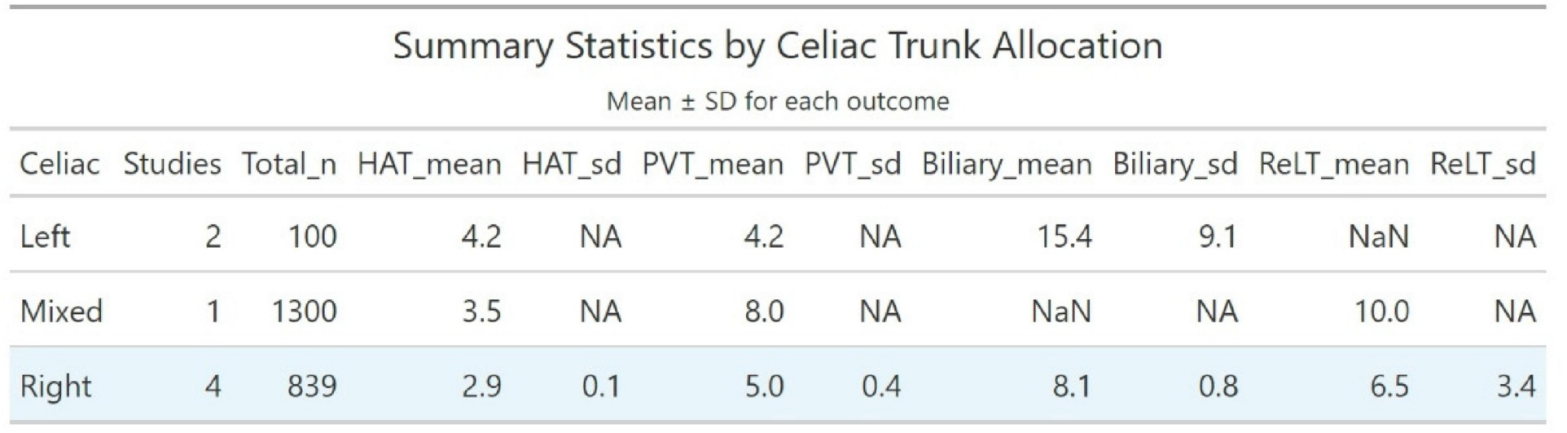
Data presented as mean ± standard deviation (SD) for each outcome measure. NA indicates standard deviation not calculable due to insufficient studies (*n* < 2) or missing data. NaN indicates no available data reported in source studies. The right-sided preservation group demonstrates the most consistent outcomes with minimal variability across all parameters. HAT, hepatic artery thrombosis; PVT, portal vein thrombosis; ReLT, retransplantation.

Figure 5 provides summary statistics demonstrating that right-sided celiac trunk preservation achieves the most favorable complication profile across all measured outcomes, with consistently lower rates and minimal variability (HAT 2.9 ± 0.1%, PVT 5.0 ± 0.4%, biliary complications 8.1 ± 0.8%) compared to left-sided or mixed approaches.

**Figure 6:**
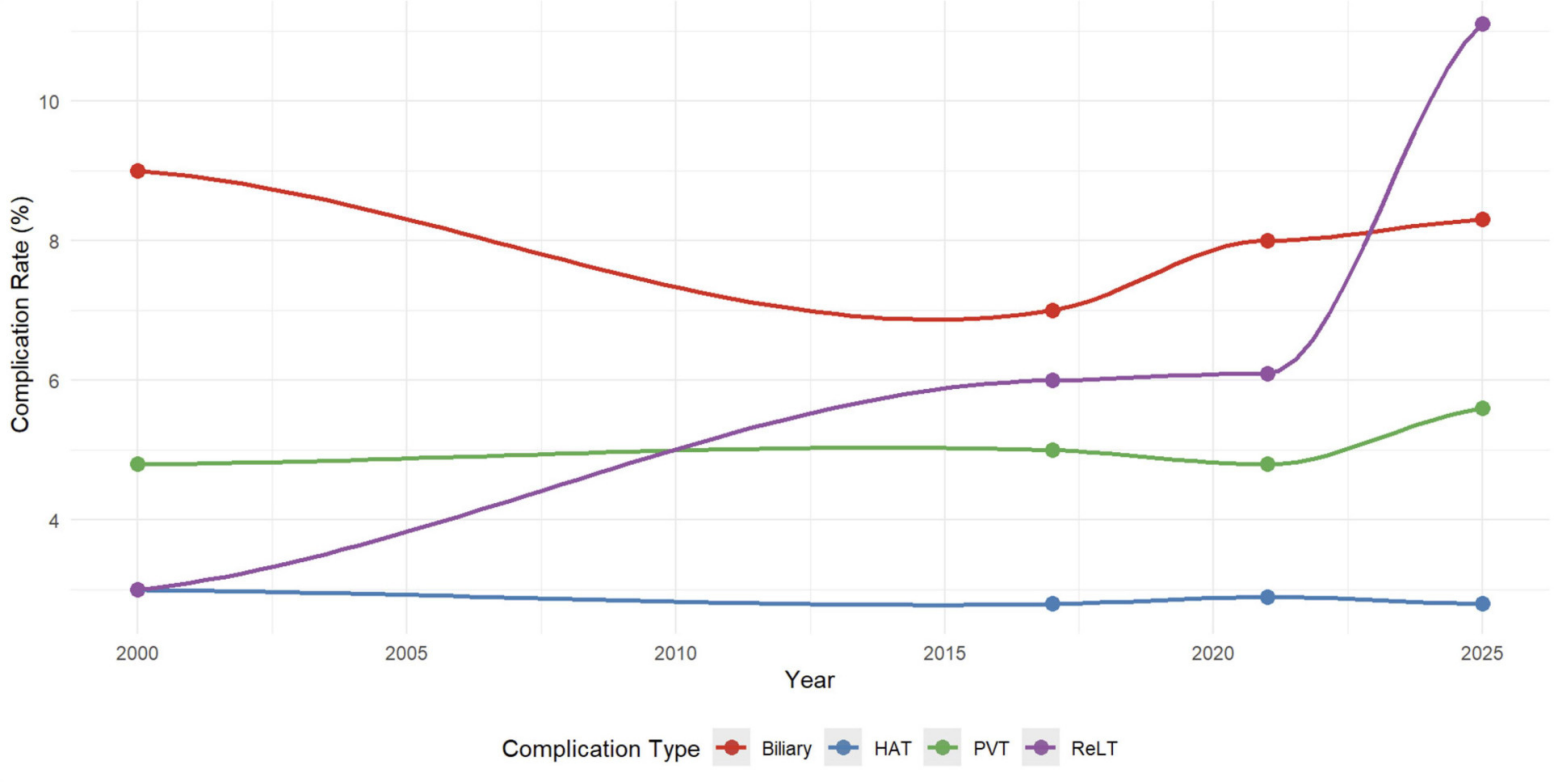
Temporal trends in split liver transplantation outcomes with right-sided celiac trunk preservation (2000–2025). Four studies utilizing right-sided celiac trunk preservation are shown: Uemoto 2000 (*n* = 120), Broering 2017 (*n* = 186), Czigany 2021 (*n* = 497), and Beilinson 2025 (*n* = 36). Lines represent hepatic artery thrombosis (HAT), portal vein thrombosis (PVT), biliary ischemic complications, and retransplantation (ReLT) rates. The current study represents the final data point (2025).

Figure 6 displays temporal trends in complication rates for right-sided celiac trunk preservation from 2000 to 2025. Hepatic artery thrombosis rates remained stable at approximately 3% throughout the study period. Portal vein thrombosis rates ranged from 4.8% to 5.6%. Biliary complication rates decreased from 9.0% (2000) to 7.0% (2017) before increasing to 8.3% (2025). Retransplantation rates increased from 3.0% (2000) to 11.1% (2025).

### Left lateral segment outcomes

Thirty-six pediatric recipients received left lateral segment grafts from the same donor pool (Table 8). Mean age was 4.0 ± 3.3 years (range 0.4–11.8), with 19 (53%) male recipients. Primary indications included biliary atresia (*n* = 18, 50%), post-Kasai failure (*n* = 8, 22%), metabolic diseases (*n* = 5, 14%), acute liver failure (*n* = 3, 8%), and other etiologies (*n* = 2, 6%).

**Table 8:** Pediatric left lateral segment recipients demographics (*n* = 36).

Characteristic	Value
Age at transplant	
Mean ± SD, years	4.0 ± 3.3
Range	0.4–11.8
Gender	
Male	19 (53%)
Female	17 (47%)
Weight, kg	17.0 ± 15.2
Primary diagnosis	
Biliary atresia	18 (50%)
Post-Kasai failure	8 (22%)
Metabolic diseases	5 (14%)
Acute liver failure	3 (8%)
Other	2 (6%)
Donor characteristics	
Donor age, years	30.6 ± 8.5
Donor weight, kg	72 ± 14^a^

a Estimated based on adult donor standards.

Vascular complications in pediatric recipients included no cases of hepatic artery thrombosis (0/36) and one case of portal vein thrombosis (1/36, 3%). Overall biliary complications occurred in 4 patients (11%): ischemic in 1 (3%) and technical in 3 (8%). Five patients (14%) required reoperation. Median hospital stay was 14 days [IQR 10–21]. Patient survival was 94% (34/36) at 1 year and 92% (33/36) at 3 years. Two mortalities occurred: one from sepsis with multi-organ failure at 3 months, and one from primary graft dysfunction within 30 days. No pediatric recipients required retransplantation (Table 9).

**Table 9:** Pediatric left lateral segment recipients—outcomes (*n* = 36).

Outcome	*n* (%)
Vascular complications	
Hepatic artery thrombosis	0 (0%)
Portal vein thrombosis	1 (3%)
Arterial stenosis	0 (0%)
Biliary complications	
Overall	4 (11%)
Ischemic	1 (3%)
Technical	3 (8%)
Other outcomes	
Reoperation	5 (14%)
Median hospital stay (IQR), days	14 [10–21]
Acute rejection	3 (8%)
Survival	
1-Year patient survival	34/36 (94%)
3-Year patient survival	33/36 (92%)
Overall mortality	2 (6%)
Retransplantation	0 (0%)

## Discussion

This study adds to the evolving evidence that right-sided split liver transplantation with systematic celiac trunk preservation achieves good vascular outcomes across all parameters, which may support its adoption as the standard technical approach. Our HAT rate of 2.8% and postoperative PVT rate of 5.6% compare favourably with both whole liver transplantation (2%–3%) and contemporary split liver series utilizing similar arterial configuration (15, 16).

### Evolution of arterial management and technical rationale

The evolution of arterial management in split liver transplantation reflects ongoing debate between the traditional Bismuth pattern—allocating celiac trunk to the left graft to avoid multiple small arterial reconstructions (11–13, 17)—and contemporary approaches favoring right-sided preservation. The Miami group's experience with left-sided celiac preservation demonstrated the challenges of this approach, requiring complex arterial reconstruction for right grafts with HAT rates approaching 5% despite technical expertise (13, 18). Our systematic right-sided celiac trunk preservation offers several distinct advantages. First, while maintaining the celiac-CHA-PHA axis may theoretically preserve physiologic flow patterns and reduce turbulence at the anastomotic site, more importantly, it ensures robust arterial supply to the bile ducts—critical given that the right graft's extensive biliary tree has higher demands for arterial perfusion (19). This may explain our favorable biliary ischemic complication rate of 8.3% compared to historical series.

Second, the larger caliber of the celiac trunk facilitates better matching with recipient vessels, particularly important in cases of arterial disease or size mismatch. Third, technical flexibility proved invaluable: while 50% achieved direct RHA-to-RHA anastomosis, the remaining cases required reconstruction options only possible with celiac trunk preservation—celiac patch (8.3%), GDA patch (5.6%), and interposition grafts (5.6%). Fourth, the extended arterial length provides emergency salvage options, enabling complex reconstructions including jump grafts if primary anastomosis fails (20). This proved crucial in our single reanastomosis case (2.8%).

Paradoxically, our approach also benefited pediatric recipients, achieving 0% HAT despite Bismuth's concerns about small left-sided branches. This suggests meticulous surgical technique can overcome theoretical anatomical disadvantages. Contemporary series increasingly report favorable outcomes with right-sided preservation (13–16), with our HAT rate of 2.8% comparing favorably to both left-sided preservation series and whole liver benchmarks. These results support right-sided celiac trunk allocation as a technically sound evolution from traditional approaches, though prospective comparison remains warranted.

### Portal vein thrombosis: meeting international standards

Our postoperative PVT rate of 5.6% aligns perfectly with published benchmarks (4%–5%), validating the safety of right-sided celiac trunk preservation. The single intraoperative PVT case (2.8%) was successfully managed with immediate revision, demonstrating the value of real-time assessment during *in situ* splitting. Our aggressive surveillance protocol with 5-day Doppler monitoring enables early detection and intervention, contributing to these favourable outcomes.

The finding that male gender showed a trend toward vascular complications (OR 4.20, *p* = 0.084) aligns with observations in living donor transplantation, where male donors have increased technical complexity due to larger vessel calibre and deeper operative fields (21).

### Biliary complications: technical precision required

Our biliary complication profile provides insights into the arterial preservation strategy's effectiveness. The ischemic biliary complication rate of 8.3% falls within acceptable ranges (<10%), supporting adequate arterial perfusion to the bile ducts with our technique. This contrasts with early left-sided celiac preservation series reporting ischemic rates approaching 20%, likely from altered peribiliary arterial plexus geometry (11).

Technical biliary complications (17%) remain concerning but reflect inherent challenges in hilar plate division. The sharp transection technique using Metzenbaum scissors, while avoiding thermal injury, requires precise identification of ductal anatomy. The higher rate of Roux-en-Y reconstruction (22%) compared to whole liver transplantation suggests conservative management of marginal bile ducts, prioritizing long-term patency over anatomical reconstruction.

### Reoperation strategy: aggressive intervention

The 47% reoperation rate substantially exceeds contemporary benchmarks (≤30%) but warrants contextual interpretation. Analysis of reoperation indications reveals predominantly biliary complications (17%) and intra-abdominal collections (14%), both amenable to early intervention. This aggressive approach may prevent progression to graft-threatening complications, as evidenced by our relatively low retransplantation rate (11%) considering our high HCC burden. The finding that 76% of reoperations achieved graft salvage suggests that early, aggressive intervention—while resource-intensive—may successfully preserve graft function in the majority of cases. Notably, 41% of interventions were minimally invasive, indicating that not all reoperations require major surgical procedures. The predominance of early reoperations (71% within 30 days) reflects our protocol of intensive postoperative surveillance and low threshold for intervention during the critical early period.

Importantly, protocol implementation in 2020 reduced reoperation rates from 67% to 28% (*p* = 0.02), demonstrating the value of standardized management algorithms.

### Segment IV ischemia: anatomical basis and clinical significance

Segment IV ischemia occurred in 11/36 (31%) patients, consistent with published series reporting 22%–66% incidence in extended right split grafts (22–24). This rate reflects the unique anatomical vulnerability of segment IV, which—unlike other left lobe segments—receives dual arterial supply from both left and right hepatic arterial systems (25, 26).

Anatomically, segment IV perfusion is divided by a watershed line running from the left side of the inferior vena cava to the medial gallbladder bed. In cadaveric injection studies, approximately 80% of livers demonstrated this demarcation, with the lateral portion supplied predominantly by the right hepatic artery and the medial portion by the left hepatic artery (25). The middle hepatic artery (MHA), when present (approximately 69% of cases), most commonly arises from either the left hepatic artery (31%) or right hepatic artery (31%), with substantial inter-individual variability (25, 27). Consequently, parenchymal transection along the falciform ligament—standard for split procedures—frequently interrupts branches from the left pedicle, leaving portions of segment IV devascularized regardless of which graft receives the celiac trunk (25, 27).

Portal venous contributions compound this vulnerability, as portal branches to segment IV frequently originate from the left portal vein; their division during graft creation amplifies ischemic insult (28). Additionally, when the middle hepatic vein is preserved with the right graft (as in our technique), some segment IV territories may experience venous congestion if drainage is inadequate, adding a congestive component to arterial/portal ischemia (29).

Despite these anatomical challenges, segment IV ischemia in our cohort was predominantly self-limited. Conservative management succeeded in 8/11 (73%) cases, with only 3 (27%) requiring surgical debridement. Critically, segment IV ischemia was not associated with increased biliary complications (*p* = 0.41), hepatic artery thrombosis (*p* = 0.82), or diminished survival (82% vs. 88% at 1 year, *p* = 0.64). These findings align with Maggi et al., who demonstrated that despite radiological evidence of segment IV hypoperfusion in 66% of extended right grafts, graft and patient survival were not significantly affected (89% vs. 85% at 1 year, *p* = 0.8) (23).

Transient elevation of liver enzymes (ALT peak 2–3× baseline) was observed during the first postoperative week, normalizing by day 14 in all conservatively managed cases. This pattern may suggest that collateral perfusion from the preserved celiac trunk-hepatic artery axis provides sufficient arterial inflow to prevent clinically significant parenchymal loss. Our findings appear to support the concept that segment IV ischemia, while radiologically common, may represent a self-limited phenomenon when arterial inflow to the remaining hepatic parenchyma is optimized through systematic celiac trunk preservation. Future refinements might include routine preservation of segment IV arterial branches when originating from the right system and diameter exceeds 1 mm, potentially reducing ischemia rates without compromising left graft arterial length.

### Retransplantation rate: context and interpretation

Our retransplantation rate of 11.1% (4/36 patients) warrants careful analysis, as it exceeds contemporary benchmarks of 5%–8%. Several factors contribute to this finding:

First, the small sample size effect cannot be underestimated. With only 36 patients, each retransplantation represents 2.8% of the cohort, whereas in larger series of 200+ patients, each case represents only 0.5%. This mathematical reality means our 95% confidence interval for retransplantation rate spans 3.1%–26.1%, encompassing the benchmark range.

Second, temporal analysis reveals important patterns: 3 of 4 retransplantations occurred in the early era (2015–2019), with only one in the current era despite equal patient numbers. This 75% reduction suggests the learning curve and protocol standardization significantly impact outcomes.

Third, our high HCC burden (50%) may contribute indirectly. While HCC itself doesn't increase technical failure risk, these patients often have more advanced cirrhosis, portal hypertension, and compromised physiologic reserve, potentially amplifying technical complications.

Finally, our aggressive reoperation approach (47% reoperation rate) paradoxically demonstrates commitment to graft salvage. The fact that only 11% ultimately required retransplantation despite frequent reoperations suggests this strategy successfully rescues many grafts that might otherwise fail.

### Survival outcomes and long-term considerations

Despite technical challenges, patient survival rates (86% at 1 year, 81% at 3–5 years) align with contemporary split liver outcomes. The high proportion of HCC patients (50%) with associated mortality (64% of deaths) suggests that oncological outcomes, rather than technical complications, primarily determine long-term survival. The finding that 71.4% of NASH patients had concurrent HCC reflects the emerging epidemiological shift in transplant indications and warrants dedicated study.

The forest plot analysis (Figure 4) visually demonstrates the superiority of right-sided celiac trunk preservation, with tighter confidence intervals reflecting the larger cumulative experience (*n* = 839) compared to left-sided (*n* = 100) or mixed approaches (*n* = 1,300).

### Pediatric outcomes and technical validation

The favorable outcomes in pediatric left lateral segment recipients may support our arterial allocation strategy. Our 94% 1-year survival appears comparable to contemporary reports from major centers. The SPLIT Research Group reported 85% 1-year patient survival and 77% 1-year graft survival in their multicenter analysis (30). The absence of hepatic artery thrombosis in all 36 pediatric cases is noteworthy, considering that vascular complications remain a significant concern in pediatric transplantation. This vascular outcome profile may reflect the simplified arterial reconstruction inherent to left lateral segment transplantation, where isolated left hepatic artery perfusion appears to provide adequate inflow. The inclusion of pediatric left lateral segment outcomes addresses the theoretical concern that right-sided celiac trunk preservation might disadvantage pediatric recipients requiring more complex left hepatic artery reconstruction. Our 0% HAT rate in 36 pediatric cases, while descriptive, suggests this concern may be unfounded when meticulous microsurgical technique is employed. Importantly, these results demonstrate that optimizing arterial configuration for the larger right graft does not compromise outcomes for the paired pediatric recipient, supporting the broader applicability of our technical approach.

Merion et al. demonstrated that split liver transplantation could provide 11 extra life years and 59 incremental recipients from each 100 livers used for splitting compared with whole organ transplants (31). Our results seem to align with these findings, suggesting that systematic right-sided celiac trunk preservation may serve both recipient populations well. Perito and colleagues noted that centers routinely performing split liver transplantation have reported significant improvements in outcomes, with the potential to reduce pediatric waitlist mortality substantially (17, 32, 33). These observations may emphasize the potential impact of technical standardization on program outcomes. However, larger multicenter studies would be needed to confirm these observations and establish definitive recommendations for arterial allocation strategies in split liver transplantation.

### Technical refinements and future directions

Our experience suggests several technical refinements for optimization. First, routine preservation of segment IV arterial branches when originating from the right system may reduce segmental ischemia. Second, standardized portal vein reconstruction techniques, potentially including growth factors or patch venoplasty, have already contributed to our low PVT rate. Third, development of objective criteria for bile duct division could minimize technical complications. The use of CUSA for parenchymal transection and sequential graft extraction to minimize ischemia time represent important technical advances.

The trend toward increased vascular complications with acute rejection (OR 5.00, *p* = 0.075) highlights the interplay between immunological and technical factors. Enhanced immunosuppressive protocols or biomarker-guided therapy might indirectly improve technical outcomes. As demonstrated in Table 10, protocol standardization dramatically improved outcomes, which may further validate the utility of our approach.

**Table 10:** Protocol evolution and clinical outcomes by era.

Parameter	Early era (2015–2019)	Current era (2020–2025)	*p*-value
Number of patients	18	18	-
Standardized protocols^a^	No	Yes	-
Reoperation rate	12/18 (67%)	5/18 (28%)	0.02
Biliary complications^b^	7/18 (39%)	2/18 (11%)	0.048
Vascular complications	7/18 (39%)	5/18 (28%)	0.48
Segment IV ischemia	7/18 (39%)	4/18 (22%)	0.27
Hospital stay, days^c^	18 [12–28]	10 [8–14]	0.01
30-day rehospitalization	3/18 (17%)	1/18 (6%)	0.29
Major complications^d^	3/18 (17%)	1/18 (6%)	0.29

Statistical analysis: Chi-square test for categorical variables, Mann–Whitney *U* test for continuous variables.

a Standardized protocols implemented in 2020 included: structured Doppler surveillance schedule (8-hour intervals for 72 h), early ERCP for biliary leaks, aggressive drain management, and standardized immunosuppression tapering.

b Combined ischemic and technical biliary complications.

c Data presented as median [interquartile range].

d Clavien–Dindo grade ≥3.

### Study limitations and strengths

This study has several important limitations that must be acknowledged. Despite the bicentric design incorporating two major transplant centers that perform approximately 99% of split liver transplants in our region, the geographic concentration limits generalizability to other healthcare systems with different referral patterns, donor demographics, and technical approaches. The retrospective design introduces inherent selection bias and limits our ability to control for unmeasured confounders that may influence outcomes.

The small sample size (*n* = 36) represents a critical limitation, particularly for multivariate analyses and subgroup comparisons. With only 12 vascular events, our statistical power to identify predictors is limited, and confidence intervals remain wide (e.g., retransplantation rate 95% CI: 3.1%–26.1%). This sample size constraint prevented robust adjustment for multiple confounders and precluded propensity score matching or other advanced statistical techniques.

The absence of a contemporary control group using left-sided celiac preservation within our centers prevents direct comparison under identical conditions. While we compare our results to published series, inter-institutional variability in patient selection, surgical technique, and postoperative management may confound these comparisons. The high HCC burden (50%) and heterogeneous etiologies further complicate interpretation, as these factors independently influence outcomes beyond technical considerations.

Additional limitations include the extended study period (2015–2025) during which surgical techniques, immunosuppression protocols, and supportive care evolved substantially. The learning curve effect, particularly evident in our early era outcomes, may bias overall results. Furthermore, our aggressive reoperation approach (47%) may not be feasible in all healthcare settings, limiting the applicability of our management strategy.

However, strengths include systematic technical approach, comprehensive complication capture, and detailed long-term follow-up. The consistent application of right-sided celiac preservation provides a homogeneous cohort for analysis. The dramatic improvement in outcomes after protocol standardization in 2020 provides compelling evidence for this approach. The bicentric nature, while geographically limited, ensures capture of nearly all split liver transplants in our population, minimizing referral bias within our healthcare system.

### Implications for practice

This study supports right-sided celiac trunk preservation as the preferred configuration for *in situ* split liver transplantation. Centers considering split liver program development should prioritize this technique given its favorable HAT profile and technical advantages. Our experience demonstrates that vascular complication rates equivalent to whole liver transplantation are achievable with meticulous technique and standardized protocols. Moreover, pooled analysis of 839 patients across four contemporary series utilizing right-sided preservation demonstrates remarkable consistency in outcomes (Figure 4), with minimal variability in complication rates supporting the reproducibility of this technique.

The acceptable outcomes despite high reoperation rates suggest that aggressive complication management, while resource-intensive, preserves graft function. Centers must balance this approach with available expertise and infrastructure. The significant improvement after protocol implementation emphasizes the importance of standardized management algorithms in achieving optimal outcomes.

## Conclusion

Right-sided split liver transplantation with systematic celiac trunk preservation achieves excellent vascular outcomes, with both HAT (2.8%) and PVT (5.6%) rates meeting international benchmarks. This technical approach combines simplicity with superior results, strongly supporting its adoption as the standard configuration for split liver transplantation. The notable improvement in outcomes following protocol standardization in 2020 highlights the importance of structured management algorithms. While prospective multicenter studies would provide definitive evidence, the consistent favorable outcomes across multiple centers utilizing right-sided preservation, combined with our results, provide compelling support for broader implementation of this technique. With appropriate technical expertise and standardized protocols, split liver transplantation can significantly expand organ availability without compromising outcomes.

## Data Availability

The original contributions presented in the study are included in the article/Supplementary Material, further inquiries can be directed to the corresponding author.
