# Supplementary material for: In situ split liver transplantation with celiac trunk allocation: technical evolution and outcomes supporting right-sided preservation
Source: Front Surg. 2026 Jan 16;12:1737518. doi: 10.3389/fsurg.2025.1737518 (PMC12868976; doi:10.3389/fsurg.2025.1737518)
Supplement: Supplementary file 1 [file Table1.docx]

**Supplementary Table S1:**

| **Category** | **n (%)** | **Timing** | **Approach** |
| --- | --- | --- | --- |
| **Total reoperations** | 17 (47%) | — | — |
| *By timing* |  |  |  |
| Early (≤30 days) | 12 (71%) | — | — |
| Late (>30 days) | 5 (29%) | — | — |
| *By intervention type* |  |  |  |
| Percutaneous drainage | 4 (24%) | Early: 3, Late: 1 | Minimally invasive |
| ERCP/biliary stenting | 3 (18%) | Early: 2, Late: 1 | Minimally invasive |
| Open surgical | 10 (59%) | Early: 7, Late: 3 | Open |
| *Outcome relationship* |  |  |  |
| Graft salvage achieved | 13/17 (76%) | — | — |
| Proceeded to retransplant | 4/17 (24%) | — | — |

Data are presented as n (%). Percentages for timing and intervention type categories are calculated from total reoperations (n=17). Early reoperations were defined as occurring within 30 days of transplantation; late reoperations occurred beyond 30 days. Minimally invasive procedures included percutaneous drainage and endoscopic retrograde cholangiopancreatography (ERCP) with biliary stenting. Open surgical procedures included laparotomy for biloma drainage, Roux-en-Y revision, abscess evacuation, ischemic segment debridement, vascular revision, and wound complications. All reoperations were unplanned, triggered by clinical deterioration or abnormal imaging findings. Graft salvage was defined as avoidance of retransplantation following reoperation.

Abbreviations: ERCP, endoscopic retrograde cholangiopancreatography; POD, postoperative day.
